# Patient and public perspectives of community pharmacies in the United Kingdom: A systematic review

**DOI:** 10.1111/hex.12639

**Published:** 2017-11-08

**Authors:** Ali M. K. Hindi, Ellen I. Schafheutle, Sally Jacobs

**Affiliations:** ^1^ Centre for Pharmacy Workforce Studies Division of Pharmacy and Optometry School of Health Sciences Faculty of Biology, Medicine and Health The University of Manchester Manchester UK

**Keywords:** community pharmacy, health services, patient and public users, public health services, systematic review

## Abstract

**Background:**

The United Kingdom has been at the forefront of enhancing pharmacist roles and community pharmacy services, particularly over the past decade. However, patient and public awareness of community pharmacy services has been limited.

**Objective:**

To identify and synthesize the research literature pertaining to patient and public perspectives on: existing community pharmacy services, extended pharmacist roles and strategies to raise awareness of community pharmacy services.

**Search strategy:**

Systematic search of 8 electronic databases; hand searching of relevant journals, reference lists and conference proceedings.

**Inclusion criteria:**

UK studies investigating patient or public views on community pharmacy services or pharmacist roles from 2005 to 2016.

**Data extraction and synthesis:**

Data were extracted into a grid and subjected to narrative synthesis following thematic analysis.

**Main results:**

From the 3260 unique papers identified, 30 studies were included. Manual searching identified 4 additional studies. Designs using questionnaires (n = 14, 41%), semi‐structured interviews (n = 8, 24%) and focus groups (n = 6, 18%) made up the greatest proportion of studies. Most of the studies (n = 28, 82%) were published from 2010 onwards and covered perceptions of specific community pharmacy services (n = 31). Using a critical appraisal checklist, the overall quality of studies was deemed acceptable. Findings were grouped into 2 main themes “public cognizance” and “attitudes towards services” each with 4 subthemes.

**Discussion and conclusions:**

Patients and the public appeared to view services as beneficial. Successful integration of extended pharmacy services requires pharmacists’ clinical skills to be recognized by patients and physicians. Future research should explore different approaches to increase awareness.

## 1 INTRODUCTION

Health‐care organizations find themselves facing new challenges to keep up with growing health‐care demands of the public.^1^ Many of these challenges are associated with improving the economical, humanistic and clinical outcomes for individuals with long‐term conditions.^1^, ^2^ These demands have led policymakers to seek alternative ways to optimize the health‐care system and enhance patient care. It has been argued that for health‐care services to be more efficiently utilized, all members of the health‐care team need to collaborate and reassess their roles and contributions.^3^ Better collaboration could reduce medical errors and improve patient outcomes^4^ by combining skills, expertise and resources.^5^ Community pharmacies are part of the primary care system, yet there has been scepticism about pharmacist collaboration with other health‐care professionals due to their isolated roles and the commercial environments in which they operate.^6^, ^7^, ^8^ Yet the importance of such integration, particularly with physicians, has now been recognized.^9^

In recent decades, the advancement of the pharmacy profession has seen a movement away from a traditional supply function towards more clinically orientated activities.^10^ Community pharmacies are the most frequently visited health‐care destinations,^11^, ^12^ leading policymakers to recognize the importance of extending community pharmacists’ roles to meet growing public demands.^11^, ^13^, ^14^ This has led to novel reimbursement structures being implemented across many health‐care systems, including Australia, Canada, New Zealand and the United States,^15^, ^16^ which fund cognitive services as well as medicines supply. The United Kingdom was at the forefront of these developments, with a revised community pharmacy contractual framework being implemented in England and Wales in 2005 and Scotland in 2006, introducing funded clinical, medication review and public health services.

In England, the revised contracts specify 3 levels of service. Essential services cover traditional roles such as dispensing medications/appliances, repeat dispensing and signposting whilst advanced services focus on pharmacist medication reviews. Locally commissioned services include a wide range of medication and public health services, such as minor ailments (assessment and management of minor ailments by pharmacists), smoking cessation, lifestyle advice, emergency hormonal contraception, substance misuse, screening and vaccinations.^12^, ^15^ Moreover, consultation rooms became mandatory and subject to certain requirements ensuring patient privacy and confidentiality.

In England, there are 2 medication review services, medicines use reviews (MUR) and new medicine service (NMS). Both aim to improve patient understanding and adherence to regular or newly prescribed medication through pharmacist consultations.^17^ Similar MUR services exist in Wales^18^ and Northern Ireland;^19^ Wales also has a Discharge Medicines Review.^20^ In Scotland, pharmacists develop and prioritize pharmaceutical care plans as part of the Chronic Medication Service.^21^ Comparable services have been developed in other countries such as the medication therapy management in the United States,^22^ MedsCheck in Canada and Australia,^23^ Home Medicines Review in Australia^24^ and Long Term Conditions service in New Zealand,^25^ all of which focus on improving patient medication outcomes through pharmacist consultations.

Whilst evidence has demonstrated positive outcomes from community pharmacy services,^26^, ^27^, ^28^, ^29^, ^30^ uptake^31^, ^32^, ^33^ and awareness^12^, ^34^, ^35^, ^36^, ^37^ of some of these services have been low. A systematic review of uptake and patient outcomes of remunerated pharmacy services revealed low uptake despite improved clinical and financial outcomes,^16^ but this study did not explore patient perspectives. Two systematic reviews covering patient and public views on public health services provided in community pharmacies reinforced low awareness. The earlier review covered publications from 1990 to 2002,^34^ including studies preceding the revised UK contractual framework; the other review covered 2001‐2010.^12^ However, only 9 UK studies reported patient perspectives, and only 4 were within the time frame of the revised pharmacy contract (2005 onwards). All of the above‐mentioned reviews focussed on particular service(s) rather than community pharmacy more generally. So insight into reasons for low public and patient awareness and uptake remains limited, yet understanding patient views is important to ensure optimal design and provision of community pharmacy services that meet patients’ needs and public expectations.

The UK government has recently announced new plans to innovate community pharmacy services by introducing reward systems for high‐quality services, integration funds to improve health‐care collaboration and expanding current services to those who need urgent care.^38^ In the light of this push to further extend community pharmacy services, it is important to identify how patients and the public view community pharmacy services and pharmacist roles, and whether these have changed due to recent policy changes.

The aim of this article is to review current evidence on patient and public perspectives regarding existing community pharmacy services, extended pharmacist roles and strategies to raise awareness of community pharmacy services in the United Kingdom.

## 2 METHODS

The development and reporting of this review followed the key principles for systematic reviews.^39^ Based on the recommendations by Mays et al^40^ for synthesizing disparate evidence to inform health‐care policymaking, a narrative synthesis was undertaken, to provide a descriptive account of both qualitative and quantitative findings.

### 2.1 Definitions

In this review, the term “patients” refers to participants who have experienced the service(s) examined whilst “public” refers to participants who may not have experienced service(s).

### 2.2 Information sources and search

Eight academic databases (EMBASE, PubMed, Scopus, Web of Science, International Pharmaceutical Abstracts (IPA), Science Direct, The Cumulative Index to Nursing and Allied Health Literature (CINAHL) and PsycINFO) were searched for UK literature published from 2005 to 2016. Search terms were developed by all authors (see Table 1). Specific search strategies for each database are provided in Appendix S1. Alterative screening techniques were also employed: reference lists of included studies were scanned and relevant abstracts from UK pharmacy practice conferences published since 2005 scrutinized to explore whether they had been followed up with a full paper publication. The search took place in November/December 2016.

**Table 1:** Search terms used for the review

Concept	Search terms
Perspectives	Perspective(s) OR awareness OR “patient preference(s)” OR view(s) OR access OR opinion(s) OR perception(s)
AND patient/public	Patient(s) OR public(s) OR “service user(s)” OR consumer(s) OR customer(s) OR client(s)
AND community pharmacy	“community pharmacy” OR “community pharmacies” OR “community pharmacist(s)”
AND Service	Service(s) OR “pharmaceutical care” OR “public health” OR “medication therapy management” OR “long term care”

### 2.3 Data screening

Titles and abstracts were initially screened against the inclusion/exclusion criteria by the lead author (see Table 2). One or both of the co‐authors were consulted where queries arose. Subsequent screening involved full‐text application of the inclusion/exclusion criteria.

**Table 2:** Inclusion and exclusion criteria

Inclusion criteria	Exclusion criteria
Setting: Community pharmacy	Setting: Hospital pharmacy, outpatient pharmacy
Location: United Kingdom	Location: Outside the United Kingdom
Design/Study type: Qualitative and quantitative studies. Randomized control trials (RCTs) with a primary component eliciting participants’ perspectives on the service	Design/Study type: All types of review studies. Randomized control trials (RCTs) with no secondary aim eliciting participants’ perspectives on the service
Publication type: Peer‐reviewed journal papers	Publication type: non‐peer‐reviewed papers and conference abstracts
Publication date: 2005‐2016	Publication date: Before 2005
Focus of study: Studies exploring patient/public views on any community pharmacy service and/or extended pharmacist role that may take place in community pharmacy settings (ie independent prescribing)	Focus of study: Studies only exploring health‐care professionals or administrators’ views on pharmacy services and/or studies only assessing clinical outcomes (not eliciting views/opinions)

### 2.4 Data extraction and synthesis

Data from all included articles were extracted by the lead author and then reviewed with co‐authors. The data extracted were collated via a grid to summarize study characteristics (see Table 3): author(s), year of publication, study design, number of participants, type of pharmacy service(s), study aim, key findings and themes. Themes were identified using the following steps: Findings that demonstrated commonality were combined under a potential theme. Potential themes were compared among all studies to identify trends and patterns. Themes demonstrating a trend were collated and further analysed to interpret underlying meanings which were labelled as initial themes and subthemes. The formation of an initial thematic map adapted from Braun and Clarke^41^ was used to evaluate the strength and uniqueness of initial themes/subthemes. Any subthemes/themes that were not unique were added to broader subthemes/themes (see Figure 2).

**Table 3:** A summary table of all studies included in this review

Author(s) & year	Study design	Number of participants	Pharmacy service	Brief description of the study aim	Key findings	Theme(s) & subtheme(s)
Baraitser et al 2007^71^	Mixed methods (surveys and semi‐structured interviews)	80 clients completed questionnaires and 24 were interviewed	Chlamydia testing	Evaluate the feasibility and acceptability to users and pharmacists for chlamydia testing in independent community pharmacies	Most clients heard about the service from the pharmacist when requesting emergency contraception, and 16% (n = 13) would not otherwise have been tested. 80% of questionnaire respondents were “very satisfied” and 14% were “satisfied.” All felt “very comfortable” or “comfortable” discussing sexual health with the pharmacist. Clients valued the speed and convenience of the service and the friendly, non‐judgemental approach of the pharmacist. Confidentiality when asking for the service at the counter was suboptimal.	Public cognizance: awareness and use of pharmacy services Attitudes towards services: perceived impact, facilitator, barriers
Tinelli et al 2007^43^	Questionnaire at baseline and follow‐up of RCT	1232 patients at baseline and 1085 at follow‐up	Medicines management service	To asses patient satisfaction with, attitudes towards, and expectations of or experiences with community pharmacy in general, and to evaluate the effect of community pharmacy‐led management service on these factors	The respondents indicated that they wanted pharmacists to provide dispensing, medications review, advice on medications and health, private consultation areas, and short visit times. At follow‐up, intervention patients were more likely than control patients (*P* < .01) to rate the service provided by their pharmacist with a higher level of satisfaction, and most intervention patients stated a preference for seeing their physician to discuss their medications, although this was less marked than in control patients (76% vs 85%; *P* < .01).	Public cognizance: physicians’ authority Attitudes towards services: service vs non‐service users, perceived impact
Bissell et al 2008^57^	Semi‐structured interviews	49 patients	Medicines management service	To describe patients’ experiences of a medicines management service provided by community pharmacists for people with coronary heart disease.	Majority of patients reported positive experiences with pharmacist consultations. In particular, respondents reported deferring less to the pharmacist than they would have done to their doctor. Findings suggest that although patients cautiously welcomed the opportunity to consult with a pharmacist about their medicines, they had reservations about them making recommendations about treatment, and many still regarded the doctor as the health professional “in charge” of their medicines.	Public cognizance: perceptions of pharmacists, physicians’ supremacy Attitudes towards services: perceived impact, facilitators
Stewart et al 2008^44^	Questionnaire	103 patients	Pharmacist prescribing	To explore patients’ perspectives and experiences of pharmacist supplementary prescribing in Scotland.	89.3% of patients agreed/strongly agreed that they were satisfied with the consultation. Most patients were positive in their attitudes, agreeing that they would recommend a pharmacist prescriber to others and that they had trust in the pharmacist. However, 65% would prefer to consult a doctor.	Public cognizance: perceptions of pharmacists, physicians’ supremacy Attitudes towards services: perceived impact
Stewart et al 2009^58^	Semi‐structured interviews	18 patients	Pharmacist prescribing	Explore the perspectives of pharmacist supplementary prescribers, their linked independent prescribers and patients, across a range of settings in Scotland	Generally, patients were supportive of pharmacists as supplementary prescribers. Although patients raised no concerns, they had little idea of what to expect on their first visit, leading initially to feelings of apprehension. They praised the quality and extent of discussion relating to their medicines. All were satisfied with the service and trusted the pharmacist.	Public cognizance: awareness and use of pharmacy services, perceptions of pharmacists Attitudes towards services: perceived impact, facilitators.
Tinelli et al 2009^75^	Discrete choice experiment	204 patients	Increased pharmacist role in drug therapy management	To investigate patients’ preferences for an innovative combined prescribing‐and‐dispensing role for pharmacists in the management of drug therapies, compared to the more traditional dispensing‐only role	Respondents preferred their “current” service to either the proposed combined prescribing‐and‐dispensing role or a dispensing‐only service. Quality of treatment and cost were most influential attributes for choice of service and to a lesser extent waiting times.	Attitudes towards services: perceived impact, facilitators
Dhital et al 2010^59^	Semi‐structured interviews	102 service users	Alcohol screening and brief interventions	To investigate potential barriers and enablers of pharmacy alcohol screening and brief interventions	Accessibility and anonymity were reported as positive aspects and concerns were expressed about lack of privacy, time and whether pharmacists were knowledgeable or had suitable training to conduct screening and brief interventions.	Public cognizance: perceptions of pharmacists Attitudes towards services: facilitators, barriers
Hobson et al 2010^60^	Semi‐structured interviews	18 patients	Pharmacist prescribing	To explore the opinions of patients on the development of non‐medical prescribing	It was apparent that participants’ awareness of the training and knowledge of pharmacists was low. Some comments were made which suggest that the pharmacist is not held in very high regard by some people. Participants expressed concerns about clinical governance, privacy and whether sufficient space was available to provide the service in community pharmacies. Participants acknowledged the expert drug knowledge of pharmacists and their accessibility.	Public cognizance: awareness and use of pharmacy services, perceptions of pharmacists. Attitudes towards services: facilitators, barriers
Krska et al 2010^45^	Questionnaire	177 members of the public	Weight management	To determine the public's views on weight‐management services, including pharmacies as a potential venue, and the extent of current pharmacy involvement	There was greater awareness of commercial weight‐management clinics than of NHS‐led initiatives. Pharmacies and pharmacists were not favoured as sources of advice on weight management.	Public cognizance: awareness and use of pharmacy services, perceptions of pharmacists
Krska & Morecroft, 2010^46^	Questionnaire	300 healthy adults	Public health	To determine the views of healthy adults on the importance of activities aimed at improving public health, on the role of community pharmacies in contributing to these and the range of potential pharmacy‐based public health services	Only 23% considered that pharmacies were the best place from which to seek general health advice, irrespective of frequency of pharmacy use. About 49% of respondents considered general practitioners to be the best source of public health advice, but 23.0% selected pharmacies. There was a general lack of awareness of pharmacy capacity and role in public health. However, most supported the provision of specific services by pharmacies, especially among frequent pharmacy users. Access and long opening hours were the main facilitating factors mentioned, together with pharmacist knowledge. A significant proportion of respondents said they would not use pharmacy as a source of public health advice, due to issues around confidentiality, privacy, space and busyness.	Public cognizance: awareness and use of pharmacy services, perceptions of pharmacists, physicians’ supremacy Attitudes towards services: service vs non‐service users, facilitators, barriers
Mackridge et al 2010^65^	Focus groups	20 problematic drug users	Problematic drug users	To qualitatively explore the feasibility and desirability of further developing community pharmacy services to meet the wider health needs of problematic drug users	Many of the service users in the study were not aware of services beyond needle and syringe programmes and substitution therapy. Many service users perceived existing services to be suboptimal especially with regard to privacy, as a major concern. Good rapport between users and regular staff was highlighted as an important factor in good quality services. Pharmacies were consistently identified as having key opportunities to make useful health interventions within a range of therapeutic areas. The most widely supported roles were based around information provision and signposting.	Public cognizance: awareness and use of pharmacy services, perceptions of pharmacists, Attitudes towards services: perceived impact, facilitators, barriers
Stewart et al 2011^47^	Questionnaire	105 patients	Pharmacist prescribing	To evaluate views of patients across primary care settings in Great Britain who had experienced pharmacist prescribing	The vast majority agreed/strongly agreed that they were totally satisfied with their consultation and confident that their pharmacist prescribed as safely as their general practitioner (GP). Pharmacists were considered approachable and thorough, and most would recommend consulting a pharmacist prescriber. A small minority felt that there had been insufficient privacy and time for all their queries to be answered.	Public cognizance: perceptions of pharmacists, physicians’ supremacy Attitudes towards services: perceived impact, facilitators, barriers
Taylor et al 2012^48^	Questionnaire	97 service users and 261 non‐service users	Cardiovascular screening	To determine whether pharmacy‐based cardiovascular disease (CVD) screening reached the desired population, the local population's awareness of pharmacy screening and the views of service users and the general public about CVD screening.	The overall majority of service users (99.7%) had a positive experience of the screening service, agreeing that they were given enough time and pharmacists made them feel at ease. Perceived concerns about confidentiality and lack of privacy were among barriers identified to taking up screening. Only 9% of non‐users were aware of the pharmacy service. Significantly more service users (90.7%) agreed that a pharmacy was a good place for screening compared to the non‐users (77.4%; *P *<* *.005).	Public cognizance: awareness and use of pharmacy services Attitudes towards services: service vs non‐service users, perceived impact, facilitators, barriers
Gidman and Cowley, 2013^67^	Focus groups	26 members of the public	Pharmacy services in general	To understand the public's opinions and experiences of pharmacy services.	Participants made positive comments about pharmacy services although many preferred to see a general practitioner (GP). Participants discussed using pharmacies for convenience, often because they were unable to access GPs. Pharmacists were perceived principally to be suppliers of medicine, although there was some recognition of roles in dealing with minor ailments and providing advice. The pharmacy environment and retail setting were not considered to be ideal for private health‐care consultations.	Public cognizance: awareness and use of pharmacy services, perceptions of pharmacists, physicians’ supremacy Attitudes towards services: facilitators, barriers
Latif et al 2013^74^	Multimethod (observations and semi‐structured interviews)	Observations of 54 patient‐pharmacist MURs consultations and subsequent interviews with 34 patients.	MUR	To describe patients’ perspective of the MUR service and their understanding of the value that they derive from it.	When describing interactions with the pharmacist, participants’ expectations did not extend to having private “sit down” discussions either about medicines or any other health‐related matter. All patients reported feeling comfortable speaking with the pharmacist, who they saw as a knowledgeable expert on medicines. They appreciated the time spent with them in a private consultation. The MUR provided patients with reassurance about their medicines. Participants considered that authority over their prescribed medicines rested with their GPs or specialist prescriber.	Public cognizance: awareness and use of pharmacy services, perceptions of pharmacists, physicians’ supremacy Attitudes towards services: perceived impact, facilitators.
Maclure et al 2013^50^	Questionnaire	1855 members of the public	Pharmacist prescribing	To explore the views of the Scottish general public on non‐medical prescribing.	Views expressed by many indicated a lack of awareness of the health‐care professional's training. Some noted that non‐medical prescribers should only prescribe medicines within their competence and appropriate to their fields of practice. Respondents perceived the key benefit of non‐medical prescribing to be enhanced patient convenience arising from reduced appointment times. Many voiced concerns around the need to access patients’ medical notes prior to any prescribing decisions or actions being taken.	Public cognizance: perceptions of pharmacists, physicians’ supremacy Attitudes towards services: facilitators, barriers
Twigg et al 2013^68^	Focus groups	25 patients	Diabetes	To examine patient perspectives on the current and future roles of the community pharmacist in the management of type 2 diabetes	All participants identified that the primary expertise of the community pharmacist as medicines supply and advice regarding over‐the‐counter preparations and the interactions with their prescribed medicines. There were differing views about how much further the pharmacist's role extended to advising on prescription medicines and diseases advice. However, even those participants who identified the pharmacist as their first port of call would not necessarily act on advice without first confirming it with their doctor. More experienced participants saw pharmacist as an easy and convenient alternative when GP hard to access. Participants still had concerns about the pharmacy being somewhere they would be willing to discuss private medical problems.	Public cognizance: awareness and use of pharmacy services, perceptions of pharmacists, physicians’ supremacy Attitudes towards services: perceived impact, facilitators, barriers
Anderson & Thornley, 2014^51^	Questionnaire	921 patients	Flu vaccination	To explore the prevalence of people paying for vaccination services at community pharmacy services and why they choose to do it.	921 patients completed a survey in the 13 pharmacies selected. Of these, 199 (22%) were eligible to get their flu vaccination for free. Of the 199 patients who were eligible for free treatment, 100 (50%) had been contacted by their GP surgery to go for their vaccination, but had chosen not to go. Reasons given include accessibility, convenience and preference for pharmacy environment. Only 1% visited pharmacy in general (12/921) due to trust in the pharmacist.	Public cognizance: perceptions of pharmacists. Attitudes towards services: facilitators
Fakih et al 2014^52^	Questionnaire	215 women in Nottingham, UK, and 395 in Victoria, Australia	Weight loss treatment	To compare women pharmacy consumer experiences with weight loss treatment between Victoria, Australia, and Nottingham, UK.	The majority of women (n = 334/436) felt comfortable receiving advice from pharmacists. In the logistic regression analysis women in Nottingham were found to be significantly less likely to have utilized a pharmacy weight‐management programme in the last 5 years (OR: 0.23 CI: 0.08, 0.63) and were significantly less likely to want an ideal weight‐management programme located in a pharmacy (OR: 0.49 CI: 0.30, 0.82) compared to women in Victoria. Women who had sought a pharmacist's advice on health, in the last 12 months, were significantly more likely to want a pharmacist in their ideal weight‐management programme (OR: 2.29 CI: 1.35, 3.90)	Public cognizance: awareness and use of pharmacy services. Attitudes towards services: service vs non‐ service users, facilitators
Hill et al 2014^49^	Questionnaire	86 service users	Pharmacist prescribing in addiction services	To establish the efficacy or accessibility of pharmacist prescribing among stakeholders and service users	Patients were very pleased with the use of pharmacist prescribing clinics. When asked to rate the pharmacist's prescribing capability, 80 patients (93%) rated a 5 (very satisfied). The majority of patients agreed it was easy to make appointments, they were given enough time at appointments, and they were given more information than at their previous clinics. Several patients would have liked more privacy at their clinics, especially in community pharmacies.	Public cognizance: perceptions of pharmacists. Attitudes towards services: perceived impact, facilitators, barriers
Krska and Mackridge, 2014^72^	Mixed‐methods study (survey, nominal group technique and telephone interviews)	150 members of the public completed questionnaires, 3 members of the public attended the nominal group technique, and 10 service users were interviewed	Alcohol screening and brief advice	To explore the views of the general public and other stakeholders towards pharmacy‐based alcohol screening and advice services	The general public viewed pharmacy‐based alcohol screening services as acceptable and feasible. Privacy was the main concern of the public, but 80% were comfortable discussing alcohol in a pharmacy. Ten service users interviewed all considered the experience positive and all would recommend the service, but most wanted the service to be delivered in a private area.	Attitudes towards services: perceived impact, facilitators, barriers
Lowrie et al 2014^61^	Semi‐structured interviews	65 patients	Heart failure service	To explore and portray in detail, the perspectives of patients receiving, and pharmacists delivering an enhanced, pay for performance community pharmacy HF service.	Patients were comfortable discussing symptoms and medicines with pharmacists; they identified pharmacists as fulfilling roles that were needed but not currently addressed. Patients reported the service helped them to enact HF medicines and HF self‐care management strategies. Some used the pharmacist as a first port of call, to help decide whether to self‐refer to a GP.	Public cognizance: physicians’ supremacy Attitudes towards services: perceived impact, facilitators
Saramunee et al 2014^69^	Focus groups	16 members of the general public	Public health services	To explore experiences and views of 4 groups of participants, the general public, PHs, general practitioners (GPs) and other stakeholders (STs) on pharmacy‐based public health services, and identify potential factors affecting service use.	Accessibility and convenience were the advantages agreed by most participants. Barriers that could inhibit service utilization are perceptions of the general public towards pharmacists’ competencies, privacy and confidentiality in pharmacies, high dispensing workload, and inadequate financial support. There was agreement among all participant groups that pharmacy‐based public health services lacked publicity. A variety of promotional techniques were mentioned as potentially useful, including posters/leaflets, media advertising and recommendation by GPs.	Public cognizance: awareness and use of pharmacy services, promotional strategies, perceptions of pharmacists. Attitudes towards services: facilitators, barriers
Tucker & Stewart, 2014^62^	Semi‐structured interviews	25 patients	Skin problems	Explore the reasons why patients with undiagnosed skin problems seek advice at pharmacies	Key themes around choice of pharmacy were convenience of professional advice, triage to general practitioner (GP) care if warranted, inaccessibility of GP care and perceived non‐serious nature of the condition. Interviewees also described high levels of trust in their pharmacists. Few concerns centred on lack of privacy and the potential for misdiagnosis. Almost all participants felt positive about their pharmacy care and would revisit for future skin problems.	Attitudes towards services: perceived impact, facilitators, barriers
Fitzgerald et al 2015^54^	Questionnaire	1573 members of the public	Alcohol interventions	Determine the Scottish general public's views regarding the role and involvement of community pharmacists in reducing alcohol consumption	More than half (56.4%, 888) agreed that pharmacists could advise on safer alcohol consumption. Those agreeing expressed high levels of support (70% agreement) for all activities. However, 78% of respondents preferred to discuss issues with doctors other than pharmacists. There was a high level of agreement of trust that pharmacists would discuss issues confidentially (68.7%, 1080), with a similar proportion (64.3%, 1011) agreeing that they would be concerned over privacy in a community pharmacy.	Public cognizance: perceptions of pharmacists, physicians’ supremacy Attitudes towards services: facilitators, barriers
McCann et al 2015^66^	Focus groups	34 patients	Pharmacist prescribing	The aim of the study was to explore patients’ perspectives of pharmacists as prescribers.	There was an overwhelming lack of awareness of pharmacist prescribing. Patients discussed the importance of a multidisciplinary approach to their care and recognized limitations of the current model of prescribing. They felt that the doctor and pharmacist had varied yet complementary skills, all of which contributed to their overall care. The majority of participants could not think of any disadvantages to having a pharmacist prescribe for them, with the exception of concerns over responsibility and being limited to one area. Patients were generally very positive about this form of practice.	Public cognizance: awareness and use of pharmacy services, perceptions of pharmacists, physicians’ supremacy Attitudes towards services: perceived impact, facilitators, barriers
Saramunee et al 2015^73^	Mixed methods (surveys followed by a focus group discussion)	908 public members completed questionnaires and 5 participants in the focus group discussion	Cardiovascular public health services	To explore the experience of and willingness to use 7 pharmacy public health services related to cardiovascular risk among the general public in England	Few respondents (2.1‐12.7%) had experienced any of the 7 pharmacy public health services. Frequent service users were more likely to use services. Focus group discussions identified barriers to service use; for example, frequent staff changes, seeing pharmacist as medicines suppliers and concerns about competence for these services.	Public cognizance: awareness and use of pharmacy services, perceptions of pharmacists, physicians’ supremacy Attitudes towards services: service vs non‐service users, barriers
Wood et al 2015^70^	Focus groups	25 people aged ≥ 65 years	Community pharmacy services in general	To explore older people's opinions of current community pharmacy provision and identify potential areas for improvement.	The ability to build a trusting relationship over time was important to the people in this study. There was a general lack of awareness of the range of services available within community pharmacies, with some participants only recognizing the dispensing role. Good communication from the community pharmacy helped to improve the experience. Specific concerns included cleanliness and privacy.	Public cognizance: awareness and use of pharmacy services, perceptions of pharmacists Attitudes towards services: facilitators, barriers
Heller and Cameron, 2016^53^	Questionnaire	220 women	Injectable contraceptives	To determine the acceptability of receiving contraceptive injections from a community pharmacist	Of those 191 current non‐users, 33% (n = 64) indicated that they would consider using this method if it was available at the pharmacy. The main perceived advantages of attending the pharmacy were quicker appointments (52%) and easier access (47%).	Attitudes towards services: facilitators
Lindsey et al 2016^63^	Semi‐structured interviews	30 pharmacy users	Community pharmacy services in general	To describe how care is perceived and experienced in community pharmacies with particular focus on community pharmacy access.	The experience of developing a trusting relationship with the pharmacist is an important consideration in the context of community pharmacy accessibility. There is also a perceived lack of awareness among the general public about the extended role of community pharmacy. Participants described several experiences where they felt guilty about using the doctor for health‐care advice or to access a public health service.	Public cognizance: awareness and use of pharmacy services, physicians’ supremacy Attitudes towards services: facilitators
Michie et al 2016^64^	Semi‐structured interviews	12 women	Sexual and reproductive health services	To identify barriers and facilitators to providing interventions from pharmacies routinely	All women welcomed the interventions indicating the benefit of having different options available. They also identified possible advantages and disadvantages of each intervention. A few women questioned whether it was the role of the pharmacist to undertake contraception consultations.	Public cognizance: perceptions of pharmacists Attitudes towards services: perceived impact, facilitators, barriers
Porteous et al 2016^76^	Discrete choice experiment	1049 members of the public	Minor ailments	To determine the general public's relative preferences for community pharmacy attributes using a discrete choice experiment	When seeking help or treatment for flu‐like symptoms, respondents most valued a pharmacy service that would improve their understanding and management of symptoms, provided by staff who are trained, friendly and approachable. Waiting time, pharmacy location and availability of parking also contributed to respondents’ preferences.	Attitudes towards services: facilitators
Rodgers et al 2016^55^	Questionnaire	1000 members of the public	Medicines‐related services, particularly MUR and NMS	To compare the perceptions of pharmacists and the general public on medicines‐related services, particularly MUR and NMS services.	Few people had experienced a discussion in a private consultation room or were aware of the 2 formal services, although their willingness to use them was high. Both pharmacists and the public had high expectations that services would be beneficial in terms of increasing knowledge and understanding. People who had experienced a pharmacy service had different perceptions of pharmacists. Over two‐thirds of respondents (690) indicated that they would consider going to a pharmacy for advice if they did experience problems with a medicine. The majority of the remainder (251) indicated that they would go to their general practitioner (GP) instead.	Public cognizance: awareness and use of pharmacy services, physicians’ supremacy Attitudes towards services: service vs non‐service users, perceived impact, facilitators
Saramunee et al 2016^56^	Questionnaire	2661 members of the public	Public health services	To identify attitudes towards pharmacy characteristics and promotional methods for selected pharmacy public health services among different sectors of the general public	There were strong preferences for a pharmacy near to home or doctor's surgery and for long opening hours. Fifty percentage preferred not to use a pharmacy in a supermarket. Personal recommendation by health professionals or family/friends was reported as most likely to encourage uptake of pharmacy public health services.	Public cognizance: promotional strategies Attitudes towards services: facilitators, barriers

### 2.5 Critical appraisal

Study quality was assessed using the nine‐item checklist developed by Hawker et al^42^ for appraising disparate studies, including abstract and title, introduction and aims, method and data, sampling, data analysis, ethics and bias, results, transferability or generalizability, implications and usefulness. Critical appraisal was conducted by the lead author and results discussed with co‐authors. Each study was given a rating of “Good” (4), “Fair” (3), “Poor” (2), “Very poor” (1) for each of the 9 items. The total score (min = 9, max = 36) was used to compare quality among studies, and scores for individual items allowed insight into the contribution of components to scores. Studies were not excluded based on quality, but served to critically appraise findings.

## 3 RESULTS

### 3.1 Study selection

**Figure 1:**
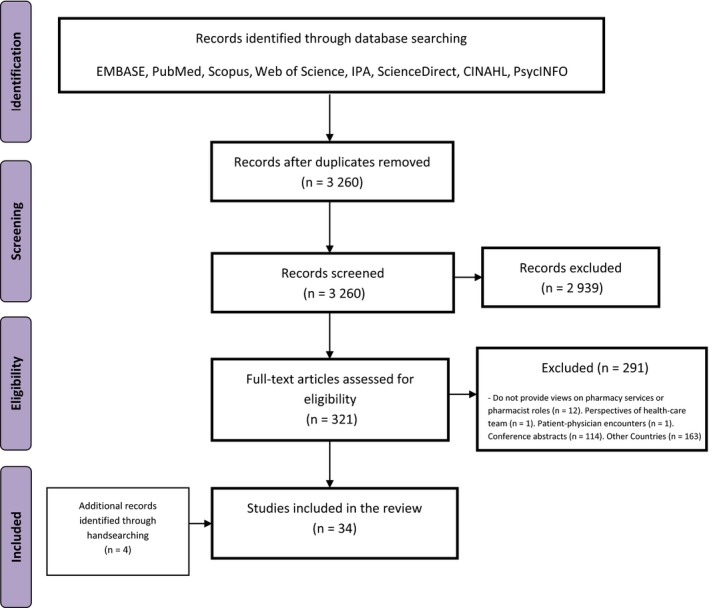
Flow diagram demonstrating the search procedure

A total of 3260 papers were identified for initial screening after duplicates were removed. Following title and abstract screening, 321 papers were assessed for eligibility via full‐text reading, with 30 studies included in the review. Manual searching of reference lists identified 4 additional studies (Figure 1).

### 3.2 Study characteristics

Study designs using questionnaires (n = 14, 41%),^43^, ^44^, ^45^, ^46^, ^47^, ^48^, ^49^, ^50^, ^51^, ^52^, ^53^, ^54^, ^55^, ^56^ semi‐structured interviews (n = 8, 24%)^57^, ^58^, ^59^, ^60^, ^61^, ^62^, ^63^, ^64^ and focus groups (n = 6, 18%)^65^, ^66^, ^67^, ^68^, ^69^, ^70^ made up the vast proportion of studies. Three mixed‐methods studies involved the use of questionnaires and semi‐structured interviews,^71^, ^72^, ^73^ one combined observations and semi‐structured interviews.^74^ The remaining 2 studies were discrete choice experiments.^75^, ^76^ Most of the studies (n = 28, 82%) were published from 2010 onwards.^45^, ^46^, ^47^, ^48^, ^49^, ^50^, ^51^, ^52^, ^53^, ^54^, ^55^, ^56^, ^59^, ^60^, ^61^, ^62^, ^63^, ^64^, ^65^, ^66^, ^67^, ^68^, ^69^, ^70^, ^72^, ^73^, ^74^, ^76^ Seven studies related to pharmacist prescribing^44^, ^47^, ^49^, ^50^, ^58^, ^60^, ^66^ which, although not exclusive to community pharmacy, provides valuable insights into opinions of pharmacists’ extended roles. Only 3 studies covered perceptions of community pharmacy services in general^63^, ^67^, ^70^ whilst the rest were service(s) specific (see Table 3).

### 3.3 Critical appraisal

Using the nine‐item checklist,^42^ the overall quality of studies was deemed acceptable (mean total score: 28.1 ± 3.4), with the lowest scoring items being ethics and bias (2.2 ± 0.6; Appendix S2). These items were evaluated based on how ethical issues were addressed and whether potential researcher biases were acknowledged. Whilst most study methods were sufficiently justified, 2 studies used surveys with questionable reliability (Cronbach α < 0.70)^44^, ^47^ and one study provided no information for their adapted questionnaire.^51^ Demographics of participants were reported by 32 studies. Two studies did not indicate why a specific sample size cut‐off number was selected.^45^, ^72^ Data analysis was not detailed in 4 surveys^44^, ^45^, ^47^, ^51^ and in one not described at all.^51^ Four qualitative studies^58^, ^59^, ^62^, ^64^ and a mixed‐methods study^72^ provided minimal details on how interviews were analysed. Most studies provided clear descriptions of findings. However, one focus group study did not provide participant quotations^65^ and another, using semi‐structured interviews, inadequately interpreted findings.^62^ Whilst all but one study mentioned ethical approval,^71^ none provided descriptions of the ethical issues such as confidentiality, sensitivity and consent. Potential biases were not reported in 18 studies,^45^, ^46^, ^48^, ^49^, ^53^, ^56^, ^57^, ^59^, ^60^, ^61^, ^64^, ^65^, ^66^, ^67^, ^68^, ^70^, ^71^, ^72^ and none of the qualitative studies acknowledged interviewer bias/reflexivity. Six studies used a small sample size/localized setting,^46^, ^48^, ^49^, ^53^, ^71^, ^73^ three inadequately represented the sample population,^44^, ^46^, ^47^ and one study was limited to one pharmacy.^51^ All but 3 studies^53^, ^62^, ^65^ reported the importance of findings to policy and practice.

### 3.4 Thematic analysis

**Figure 2:**
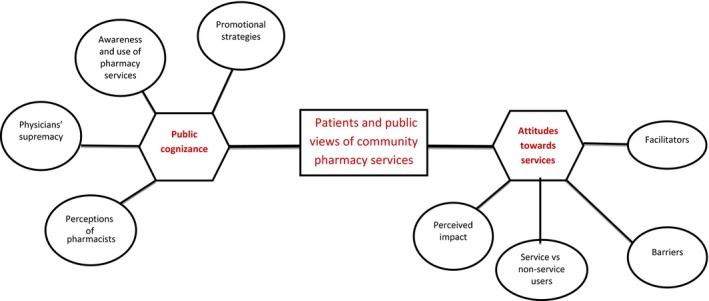
Thematic map of final themes/subthemes

Two main themes emerged: “public cognizance” and “attitudes towards services”; each with 4 subthemes (see Figure 2). These 2 themes were used to characterize patient and public views of community pharmacy services as the former provides insights into expectations from these services whilst the latter focuses on experiences of using these services. Meeting the demands of patients and members of the public requires a better understanding of both expectations and experiences.

### 3.5 Public cognizance

The theme public cognizance concerned how community pharmacies are held within the mindset of patients and the public. Public cognizance encapsulated the subthemes: “awareness and use of pharmacy services,” “perceptions of pharmacists,” “physicians’ supremacy” and “promotional strategies.” Views on pharmacies were influenced by people's awareness and use of existing pharmacy services as well as perceptions of pharmacists’ roles in health care. In addition, the comparison of pharmacists to physicians affected views of pharmacy services.

### 3.6 Awareness and use of pharmacy services

Low public or patient awareness of extended pharmacy services was a common finding.^45^, ^46^, ^48^, ^52^, ^55^, ^58^, ^60^, ^63^, ^65^, ^66^, ^67^, ^68^, ^69^, ^70^, ^71^, ^72^, ^73^, ^74^ The pharmacy setting appeared to be portrayed as a dispensary (medicines supply) and place for medicines purchase^46^ as well as advice on minor ailments.^74^ Nearly half (48.3%) of participants in one study chose dispensing as the most common reason for using pharmacies followed by the purchase of medicinal (22%) and non‐medicinal products (17.7%).^46^ In another survey assessing public views, when participants were asked how often they utilized pharmacies for certain purposes, a high proportion rated “always” for dispensing prescriptions (85.1%) and the purchase of medications (79.2%).^55^ The public and patients in qualitative studies expressed their unfamiliarity with the range of pharmacy services available^60^, ^65^, ^66^, ^67^, ^68^, ^69^, ^70^, ^72^, ^73^ whilst a survey of the general public indicated low awareness for MUR (18.2%) and NMS (8.6%) services.^55^ A study eliciting views of MUR service users discovered a mismatch between their expectations of MUR benefits and those of policymakers.^74^ In a study of patients experiencing a pharmacist prescribing service, participants indicated they were unsure about what to expect at the start of the service.^58^

Lack of awareness was accompanied by lack of exposure to, or low utilization of, pharmacy services. In a study comparing pharmacy consumers’ experiences between Australia and England, only 15.6% (24/155) of English participants had experienced a pharmacy‐based programme for weight loss,^52^ and few members of the public in another study had experienced any of 7 public health services investigated (2.1%‐12.7%).^73^ Participants in one study indicated that an influential factor for lack of awareness was pharmacy services not being publicized.^69^ In a study piloting a chlamydia testing service, the vast majority of survey respondents would not have tested, or tested elsewhere, prior to the study.^71^ Moreover, only one participant in a study preferred the pharmacy as their first choice for advice whilst 28 participants (15.8%) choose the pharmacy as the least preferred option.^45^ However, respondents in 2 surveys demonstrated willingness to use extended pharmacy services despite low initial awareness.^48^, ^73^

### 3.7 Perceptions of pharmacists

Patient and public perceptions of pharmacists seemed to influence their preferences for pharmacy‐led services. There appeared to be resistance to acknowledge the pharmacist as an essential member of the health‐care team.^46^, ^60^, ^68^, ^70^ Members of the public rated the importance of pharmacist roles with regard to certain public health issues relatively low in a questionnaire study, whilst activities linked to their traditional roles such as advice on medication usage, side‐effects and disposal were rated highest.^46^ Women in a study assessing the acceptability of pharmacies providing sexual and reproductive health services questioned whether it was the pharmacist's role to supply emergency contraception.^64^ In another, patients expressed their preferences for nurses over pharmacists due to the perception that pharmacists were “behind the counter” health‐care staff.^60^ In 2 focus group studies, pharmacist roles were perceived to be limited to dispensing and minor conditions.^68^, ^70^

Only 1% of patients in a survey chose “trust in the pharmacist” as a reason to visit the pharmacy,^68^ yet in another survey, trust in pharmacists was high.^56^ Three studies showed that patients and the public were suspicious of pharmacist commercial affiliations and financial motives.^50^, ^57^, ^67^ Moreover, concerns were identified regarding the pharmacist's lack of knowledge and training to conduct services beyond dispensing.^50^, ^67^, ^75^ However, participants in one study were supportive of pharmacists providing advice, referrals and recommendations.^54^

Despite reluctance in supporting pharmacists to carry out extended roles, their expertise in medications was acknowledged.^60^, ^73^, ^75^ Participants in pharmacist prescribing studies were generally supportive of, and confident in, pharmacist prescribing roles.^44^, ^47^, ^58^, ^66^

### 3.8 Physicians’ supremacy

Whilst the scope of these studies was specific to pharmacies, patient and public perceptions of the roles and standing of physicians influenced their views on pharmacists and pharmacy services. In survey studies, the majority of respondents preferred physicians to pharmacists regardless of the service provided.^43^, ^44^, ^46^, ^54^ In a study focusing on public health services, physicians (49%) were preferred to pharmacists (23%),^46^ whilst both the intervention and controls (76% and 84%, respectively) in an RCT investigating the effect of a community pharmacy‐led medicines management service on patients attitudes preferred physicians for health discussions and enquiries.^43^ Regarding prescribing, 65% of patients would prefer to consult a doctor^44^ and 78% of participants in another study preferred to discuss alcohol consumption issues with physicians.^54^ In contrast, the participants in one survey indicated a preference for pharmacists (69%) over physicians (25%) regarding medication advice^55^ and 97% in another survey preferred pharmacists regarding prescribing.^49^

Physicians were viewed as superior to pharmacists in knowledge and training,^50^, ^60^ and their perceived authority affected patients’ views on pharmacists making medication recommendations.^57^ This hierarchical portrayal created a division for patients between both health‐care providers, with physicians considered for diagnostic roles and more serious conditions whilst minor issues were deemed more suitable for pharmacists.^66^, ^74^ In 2 studies, this hierarchical structure led to increased accessibility of pharmacy services, as patients preferred to see the pharmacist when conditions were perceived to be not serious enough or worthy of physicians’ time.^61^, ^63^

Of particular interest was how the dynamics of the patient‐physician relationship influenced the patients’ perceived need of pharmacists. Good relationships or experiences with physicians reduced the need to seek pharmacist advice and vice versa.^68^ In one study, the physicians’ influence extended to the point that patients required physicians’ authorization or recommendation to convince/reassure them to utilize pharmacy services.^68^ In fact, members of the public in one study claimed that their trust in pharmacists would be inspired by physicians’ confidence in pharmacists.^67^ Even if the pharmacist was accessed as the first port of call, their advice required physicians’ confirmation before being acted upon.^68^ Similarly, the majority of members of the public in one study preferred a general practitioner to supervise and review pharmacist prescribing.^50^

### 3.9 Promotional strategies

Despite numerous studies showing a lack of awareness for pharmacy services, there was a shortage of studies discussing strategies that could effectively promote pharmacy services. Only 2 papers (by the same author) elicited public views regarding effective promotional schemes.^56^, ^69^ One questionnaire‐based study demonstrated word of mouth to be the most effective promotional strategy, either from a health‐care professional, or family members or friends.^56^ Focus group discussions with members of the public did not reveal specific preferences but identified various approaches that could enhance service utilization such as posters, media and physicians’ support.^69^

### 3.10 Attitudes towards services

The theme “attitudes towards services” pertained to the actual service experience and covered 4 subthemes: “service vs non‐service users,” “perceived impact,” “facilitators” and “barriers.” Patient and public attitudes were influenced by frequency of service usage and the perceived impact or benefit from using these services. Utilizing pharmacy services was associated with a number of facilitators and barriers which affected preferences for using such services.

### 3.11 Service vs non‐service users

The extent of support for pharmacy services was dependent upon the frequency of their usage by patients. More frequent service users tended to favour extended pharmacy services and revealed more support for pharmacists performing numerous different roles. Six surveys that measured this all demonstrated that patients more acquainted with the pharmacy setting were more supportive of extended pharmacy services.^43^, ^46^, ^48^, ^52^, ^55^, ^73^ Four of these studies compared more frequent users to less frequent users^46^, ^52^, ^55^, ^73^ whilst the other 2 compared service users to non‐service users.^43^, ^48^

### 3.12 Perceived impact

The majority of studies suggested that patients and members of the public perceived pharmacy services to be beneficial. With the exception of one study,^65^ most participants were positive and satisfied with pharmacy services.^43^, ^44^, ^48^, ^49^, ^57^, ^61^, ^65^, ^66^, ^71^, ^72^ Of specific interest was how services were perceived to be beneficial. Pharmacy services provided reassurance for patients in some studies.^57^, ^74^ Patients in another study believed that pharmacist discussions boosted their knowledge and confidence in medication usage which consequently enhanced future patient‐physician discussions.^61^ Another perceived benefit was that pharmacists provided assessments whether patients required visits to the physician.^61^, ^62^ In some studies, patients simply appreciated having an alternative source of information available to them.^57^, ^64^, ^68^ Open‐ended comments in a survey revealed that patients believed medicines optimization services had enhanced their appreciation of pharmacists’ knowledge and understanding.^55^ Conversely, negative remarks in some studies involved pharmacy services being perceived as unnecessary^57^ since physicians provided all the information patients needed.^74^

### 3.13 Facilitators

This subtheme focused on the features that enhanced the use of pharmacy services from the patient and public perspective. Ease of access and convenience were the most commonly mentioned advantage accredited to pharmacy services.^44^, ^46^, ^47^, ^48^, ^49^, ^50^, ^54^, ^60^, ^62^, ^65^, ^67^, ^68^, ^69^, ^70^, ^72^, ^73^, ^75^ Participants in 3 of these studies made specific reference to the non‐appointment based nature of community pharmacies.^61^, ^62^, ^63^ In 2 surveys, most respondents selected access and convenience as the main reasons for accessing pharmacy services.^51^, ^53^ The open‐ended questions in 3 other surveys^46^, ^48^, ^50^ as well as a nominal group discussion^72^ also identified access and convenience as the most advantageous aspect.

Pharmacists’ professionalism was also influential to patients’ satisfaction and was attributed to them being friendly, approachable, non‐judgemental and possessing excellent communication skills.^57^, ^66^, ^71^ Pharmacists’ mannerisms were seen as the cornerstone of building the patient‐pharmacist relationship,^63^, ^69^, ^70^ which was linked with positive perceptions and support for such services.^50^, ^60^, ^63^, ^65^, ^68^, ^69^, ^70^, ^76^ The majority of patients in 5 studies expressed comfort with having discussions with pharmacists,^49^, ^52^, ^54^, ^71^, ^72^ and 2 studies revealed that patients felt more comfortable having discussions with pharmacists compared to physicians.^44^, ^47^

Other less commonly mentioned advantages in studies were signposting and referral,^65^ service quality,^58^, ^75^ collaboration with physicians.^47^, ^50^ Older people in one study indicated preference of independent pharmacies over large pharmacy chains.^70^

### 3.14 Barriers

Studies repeatedly raised a perceived lack of privacy and confidentiality as a barrier to using extended pharmacy services,^46^, ^47^, ^48^, ^49^, ^50^, ^54^, ^60^, ^62^, ^65^, ^67^, ^68^, ^69^, ^70^, ^71^, ^72^, ^75^ with the pharmacy environment not considered an appropriate venue for private discussions. Particular concerns were about busyness and conversations being overheard, particularly regarding private matters. In addition, 2 studies revealed the specific dissatisfaction of service users with supermarket pharmacies in terms of privacy.^56^, ^68^ It was apparent that patients and members of the public were unfamiliar with consultation rooms offered at UK pharmacies since the introduction of the new community pharmacy contracts. One survey demonstrated low utilization of consultation rooms, with only 28.8% and 19.4% of respondents experiencing advice or services in private rooms, respectively.^55^ Participants in 2 further studies revealed low awareness of private consultation rooms^63^, ^68^ whilst members of the public in another study avoided using them as they associated their usage with substance misuse services.^67^ Interestingly, participants in 2 studies mentioned the lack of use or availability of private rooms.^65^, ^69^ Participants in one study argued that privacy remained an issue even when using consultation rooms.^59^ In contrast, patients in one pharmacist prescribing study were aware and reassured by the presence of private rooms.^50^

In several studies, participants perceived the pharmacist's lack of access to medical records,^50^, ^60^, ^62^, ^67^ inability to prescribe^67^ and communication difficulties with other health‐care providers^65^, ^67^, ^75^ as significant barriers to pharmacists’ extended roles in the wider health‐care system. Patients in another study mentioned pharmacists’ lack of authority to carry out extended roles.^74^ Some questioned whether pharmacists had enough time to carry out additional services due to their high workload,^66^, ^67^, ^68^, ^69^ which may lead to a lack of continuity for service provision.^69^ Lack of continuity was also raised as a barrier in another focus group study suggesting that pharmacy services were not always conducted by the same staff which reduced patient‐pharmacist rapport and confidentiality.^73^

## 4 DISCUSSION

To the authors’ knowledge, this is the first systematic review that has focused on patient and public perspectives specific to UK community pharmacy services. The past decade has seen the initiation of novel services and extended roles within a revised community pharmacy contractual framework. This review focused on gaining a deeper understanding of how these recent policy changes may have influenced patient and public views. Findings will also help to identify barriers to providing community pharmacy services which effectively meet patients’ needs, enhancing recommendations to policymakers.

The current review builds upon and extends the findings of 2 previous systematic reviews.^12^, ^34^ There was similarity with these earlier reviews in that the perceived impact of pharmacy services was high despite low awareness. However, the current review provides a detailed account of the factors facilitating the use of pharmacy services and physicians’ influence on the public's view of pharmacy services. In addition, the current review has provided evidence specific to MURs, the NMS and pharmacist prescribing, none of which have been explored previously. Furthermore, the substantial increase in qualitative studies within the last decade allowed for a comprehensive understanding and description of findings in this review.

This review focused on the United Kingdom, which was effective in reducing studies to those conducted in a single administrative and organizational context. However, this served as a possible limitation in that other countries may have provided pertinent findings, particularly ones with similar advancements in community pharmacy such as the United States, Canada, Australia and New Zealand. Another limitation was the omission of independent multiple‐author study selection and data extraction to reduce bias. However, data extraction was reviewed and discussed thoroughly with the co‐authors. Although not an exclusive community pharmacy role, the perceptions of pharmacist prescribing provided additional depth to the review and were applicable to community pharmacy services more generally.

International literature addressing the first theme, “public cognizance,” confirms that low public awareness is not exclusive to the United Kingdom.^77^, ^78^, ^79^, ^80^, ^81^, ^82^, ^83^, ^84^, ^85^, ^86^ The considerable lack of awareness of extended pharmacy services and pharmacist roles suggests that more could be done to promote the pharmacy setting as an attractive venue for health‐care delivery. Nevertheless, little has been done in the way of promoting pharmacy services or enhancing public understanding of pharmacists’ knowledge and skills.^69^, ^87^, ^88^ In relation to promotional strategies, patients in a Canadian study perceived word of mouth from pharmacy staff as the most effective method^77^ which correlated with findings in this review.^56^, ^69^ However, international literature exploring effective promotional methods is also lacking. Moreover, pharmacists’ low confidence or unwillingness to perform non‐traditional roles, together with the belief that balancing dispensing duties with extended services is unachievable,^6^, ^8^, ^12^, ^69^, ^89^, ^90^ also needs to be addressed.

Perceptions of physicians being at the top of the health‐care hierarchy were also common in other countries, including Australasia,^84^ North America,^78^ Europe^91^ and Middle East.^82^ This is borne out in evidence where physician‐pharmacist collaboration in clinical settings has been shown to significantly improve patient outcomes.^92^, ^93^, ^94^, ^95^ However, physicians have shown a reluctance to support pharmacist integration due to their unawareness of extended services.^90^, ^96^, ^97^ Moreover, physicians believe that clinical roles are better suited for themselves,^98^, ^99^ that pharmacists lack the training or ability to carry out extended roles,^6^, ^78^, ^99^ and have suspicion of pharmacists’ financial motives.^99^ Physicians have also been concerned that extending pharmacist roles and granting them more access to patient information would compromise patient confidentiality^99^ and threaten physicians’ health‐care authority.^8^, ^99^ In order to achieve better recognition and integration of physician and pharmacy services, it will be crucial to develop a better understanding of each other's knowledge, skills and potential contribution to patient care. In England, there has been a recent national initiative to introduce 490 clinical pharmacists across 650 general practice (GP) surgeries in phase 1,^100^ and a further 1500 in phase 2,^101^, ^102^ which aims to use pharmacists’ clinical skills and knowledge to relieve pressures on physicians and thus optimizing patient care.^100^, ^101^ Initial feedback from the first phase has revealed improved integration between clinical pharmacists and other members of the health‐care team.^101^

Regarding patient and public attitudes towards pharmacy services, international literature confirms findings from this review that the convenience of community pharmacies^78^, ^82^, ^85^, ^103^ and having well‐established patient‐pharmacist relationships^77^, ^78^, ^79^, ^80^, ^85^, ^102^, ^103^ are key facilitators for the usage of pharmacy services. So if it is indeed important that patients experience services in order to build rapport with their pharmacists, fully acknowledge their worth^36^ and consider recommending them to peers,^104^ then pubic promotion campaigns can only go so far. Furthermore, when designing effective community pharmacy services, it will be important to take patients’ preferences into account, where access to and convenience of pharmacy services is particularly valued.

Privacy and confidentially of the pharmacy environment, or rather the lack thereof, was considered a major barrier for using advanced pharmacy services. Moreover, patient and public awareness of private consultation rooms remains low, despite their existence being a requirement of the new community pharmacy contract. Furthermore, patients/the public perceived pharmacists’ limited authority to be a barrier for broadening roles. Whilst the introduction of pharmacist independent prescribing in 2006^105^ has gone some way to overcoming this, integration within the wider primary care team remains crucial. The introduction of “summary care records,” a scheme introduced in 2016,^106^ which permits a range of health‐care professionals, including pharmacists and pharmacy technicians, to access core clinical and medication information, with the patient's consent, may prove to be another important step towards integration and autonomy.

## 5 CONCLUSION

This systematic review provides an update of patient and public perceptions of community pharmacy services since the introduction of a revised contractual framework for community pharmacy in the United Kingdom, which introduced funding for cognitive services. Whilst the majority of literature suggests that patient and public opinions about community pharmacy services are positive, awareness of pharmacy services beyond medicines supply remains low. Patients still look to their physicians, so successful integration of pharmacy services into the primary care pathway will be essential. For this to be achieved, pharmacists’ clinical skills beyond medicines supply need to be recognized and embraced by patients and physicians alike. Furthermore, potential barriers within pharmacy, such as low pharmacist confidence, reorganization of workload to accommodate high‐quality services effectively and the potential conflict a commercial environment poses, also need to be addressed. Ease of access and convenience of pharmacies present a major advantage for their usage. Future research should explore different approaches to increasing awareness, evaluate the effectiveness of different methods of promotion and understand what the public as well as other stakeholders consider to be effective.

## References

[1] Abbing R . Health, Healthcare and ageing populations in Europe, a human rights challenge for European health systems. Eur J Health Law. 2016;23:435‐452. 29210245 10.1163/15718093-12341427

[2] England NHS . The NHS Belongs to the People: A Call to Action. London: NHS England; 2013.

[3] Department of Health . Healthy Lives, Healthy People: Our Strategy for Public Health in England. Norwich: Department of Health; 2010.

[4] Makowsky MJ , Schindel TJ , Rosenthal M , Campbell K , Tsuyuki RT , Madill HM . Collaboration between pharmacists, physicians and nurse practitioners: a qualitative investigation of working relationships in the inpatient medical setting. J Interprof Care, 2009;23:169‐184. 19234987 10.1080/13561820802602552

[5] Bridges DR , Davidson RA , Odegard PS , Maki IV , Tomkowiak J . Interprofessional collaboration: three best practice models of interprofessional education. Med Educ Online. 2011;16:6035 https://doi.org/10.3402/meo.v16i0.6035 10.3402/meo.v16i0.6035 PMC3081249 21519399

[6] Bryant LJ , Coster G , Gamble GD , McCormick RN . General practitioners’ and pharmacists’ perceptions of the role of community pharmacists in delivering clinical services. Res Social Adm Pharm. 2009;5:347‐362. 19962678 10.1016/j.sapharm.2009.01.002

[7] Adamcik BA , Ransford HE , Oppenheimer PR , Brown JF , Eagan PA , Weissman FG . New clinical roles for pharmacists: a study of role expansion. Soc Sci Med. 1986;23:1187‐1200. 3810205 10.1016/0277-9536(86)90338-2

[8] Edmunds J , Calnan MW . The reprofessionalisation of community pharmacy? An exploration of attitudes to extended roles for community pharmacists amongst pharmacists and General Practioners in the United Kingdom. Soc Sci Med. 2001;53:943‐955. 11522139 10.1016/s0277-9536(00)00393-2

[9] Royal Pharmaceutical Society . Improving Patient Care Through Better General Practice and Community Pharmacy Integration. London: Royal Pharmaceutical Society; 2015.

[10] Wiedenmayer K , Summers R , Mackie C , Gous A , Everard M , Tromp D . Developing Pharmacy Practice: A Focus on Patient Care. Geneva: World Health Organisation and International Pharmaceutical Federation; 2006.

[11] Department of Health . Pharmacy in England: Building on Strengths – Delivering the Future. London: Department of Health; 2008.

[12] Eades CE , Ferguson JS , O'Carroll RE . Public health in community pharmacy: a systematic review of pharmacist and consumer views. BMC Public Health. 2011;11:582. 21777456 10.1186/1471-2458-11-582 PMC3146877

[13] England NHS . Improving Health and Patient Care Through Community Pharmacy – A Call to Action. London: NHS England; 2013.

[14] World Health Organization . The Role of the Pharmacist in the Healthcare System. Tokyo, Japan: World Health Organization; 1997.

[15] Agomo C . The role of community pharmacists in public health: a scoping review of the literature. J Pharm Health Serv Res. 2012;3:25‐33.

[16] Houle SK , Grindrod KA , Chatterley T , Tsuyuki RT . Paying pharmacists for patient care: a systematic review of remunerated pharmacy clinical care services. Can Pharm J (Ott). 2014;147:209‐232. 25360148 10.1177/1715163514536678 PMC4212445

[17] PSNC . The relationship between the New Medicine Service and Medicines Use Reviews. http://psnc.org.uk/wp-content/uploads/2013/07/NMS_MUR_relationship.pdf. Accessed November 17, 2016.

[18] Community Pharmacy Wales . Variations between the MUR service in Wales and England. http://www.cpwales.org.uk/Contractors-Area/Pharmacy-Contact-Services/Advanced-Services/MUR-s/Variations-between-Wales-England.aspx. Accessed January 13, 2017.

[19] Health and Social Care Board . Community Pharmacy Medicines Use Review (MUR) Service Guidance for Conducting MURs. Belfast: Health and Social Care Board; 2014.

[20] NHS Wales . Changes to the medicines use review service. https://www.wcppe.org.uk/sites/default/files/file/MUR/Community%20Pharmacy%20Contractual%20Framework%20Service%20Developments%20November%202011.pdf. Accessed January 13, 2017.

[21] NHS Education for Scotland . eCMS Quick reference guide. http://www.communitypharmacy.scot.nhs.uk/documents/documentation_page/eCMS_Quick_Reference_Guide.pdf. Accessed January 12, 2017.

[22] Pellegrino AN , Martin MT , Tilton JJ , Touchette DR . Medication therapy management services: definitions and outcomes. Drugs. 2009;69:393‐406. 19323584 10.2165/00003495-200969040-00001

[23] Dolovich L , Gagnon A , McAiney C , Sparrow L , Burns S . Initial pharmacist experience with the Ontario‐based MedsCheck program. Can Pharm J (Ott). 2008;141:339‐345. e1.

[24] White L , Klinner C , Carter S . Consumer perspectives of the Australian Home Medicines Review Program: benefits and barriers. Res Social Adm Pharm. 2012;8:4‐16. 21493164 10.1016/j.sapharm.2010.11.003

[25] Central TAS . Guide to the Community Pharmacy Long Term Conditions Service, 2nd edn Wellington: Central TAS; 2014:1‐46.

[26] Armour CL , Smith L , Krass I . Community pharmacy, disease state management, and adherence to medication – A review. Dis Manag Health Outcome. 2008;16:245‐254.

[27] Cheema E , Sutcliffe P , Singer DR . The impact of interventions by pharmacists in community pharmacies on control of hypertension: a systematic review and meta‐analysis of randomized controlled trials. Br J Clin Pharmacol. 2014;78:1238‐1247. 24966032 10.1111/bcp.12452 PMC4256613

[28] Lindenmeyer A , Hearnshaw H , Vermeire E , Van Royen P , Wens J , Biot Y . Interventions to improve adherence to medication in people with type 2 diabetes mellitus: a review of the literature on the role of pharmacists. J Clin Pharm Ther. 2006;31:409‐419. 16958818 10.1111/j.1365-2710.2006.00759.x

[29] Bell S , McLachlan AJ , Aslani P , Whitehead P , Chen TF . Community pharmacy services to optimise the use of medications for mental illness: a systematic review. Aust New Zealand Health Policy. 2005;2:29. 16336646 10.1186/1743-8462-2-29 PMC1345690

[30] Blenkinsopp A , Anderson C , Armstrong M . Systematic review of the effectiveness of community pharmacy‐based interventions to reduce risk behaviours and risk factors for coronary heart disease. J Public Health Med. 2003;25:144‐153. 12848404 10.1093/pubmed/fdg030

[31] Gush A . Is funding for advanced services being misdirected? Pharm J. 2006;276:320.

[32] Hall M . Decommissioning – two areas explain the cuts. http://www.chemistanddruggist.co.uk/news/decommissioning-%E2%80%93-two-areas-explain-cuts. Accessed January 25, 2017.

[33] Collins A . ‘More needs to be done’ to increase flu uptake. http://www.chemistanddruggist.co.uk/news/sector-welcomes-second-year-flu-service. Accessed January 25, 2017.

[34] Anderson C , Blenkinsopp A , Armstrong M . Feedback from community pharmacy users on the contribution of community pharmacy to improving the public's health: a systematic review of the peer reviewed and non‐peer reviewed literature 1990‐2002. Health Expect. 2004;7:191‐202. 15327458 10.1111/j.1369-7625.2004.00274.x PMC5060240

[35] Ogunbayo OJ , Schafheutle EI , Cutts C , Noyce PR . Self‐care of long‐term conditions: patients’ perspectives and their (limited) use of community pharmacies. Int J Clin Pharm. 2017;39:433‐442. 28120168 10.1007/s11096-016-0418-y PMC5371633

[36] Elliott RA , Boyd MJ , Waring J , et al. Understanding and Appraising the New Medicines Service in the NHS in England (029/0124). Nottingham University School of Pharmacy Department of Health Policy Research Programme Project; 2014.

[37] Famiyeh IM , McCarthy L . Pharmacist prescribing: a scoping review about the views and experiences of patients and the public. Res Social Adm Pharm. 2017;13:1‐16. 26898951 10.1016/j.sapharm.2016.01.002

[38] Department of Health . New Plans to Modernise Community Pharmacies. London: NHS efficiency: Department of Health; 2016.

[39] Petticrew M , Roberts H . Systematic Reviews in the Social Sciences: A Practical Guide. Oxford: Blackwell; 2006.

[40] Mays N , Pope C , Popay J . Systematically reviewing qualitative and quantitative evidence to inform management and policy‐making in the health field. J Health Serv Res Policy. 2005;10(Suppl 1):6‐20. 16053580 10.1258/1355819054308576

[41] Braun V , Clarke V . Using thematic analysis in psychology. Qual Res Psychol. 2006;3:77‐101.

[42] Hawker S , Payne S , Kerr C , Hardey M , Powell J . Appraising the evidence: reviewing disparate data systematically. Qual Health Res. 2002;12:1284‐1299. 12448672 10.1177/1049732302238251

[43] Tinelli M , Bond C , Blenkinsopp A , et al. Patient evaluation of a community pharmacy medications management service. Ann Pharmacother. 2007;41:1962‐1970. 17971403 10.1345/aph.1K242

[44] Stewart DC , George J , Bond CM , Cunningham IT , Diack HL , McCaig DJ . Exploring patients’ perspectives of pharmacist supplementary prescribing in Scotland. Pharm World Sci. 2008;30:892‐897. 18787976 10.1007/s11096-008-9248-x

[45] Krska J , Lovelady C , Connolly D , Parmar S , Davies MJ . Community pharmacy contribution to weight management: identifying opportunities. Int J Pharm Pract. 2010;18:7‐12. 20405590 10.1211/ijpp.18.01.0003

[46] Krska J , Morecroft C . Views of the general public on the role of pharmacy in public health. J Pharm Health Serv Res. 2010;1:33‐38.

[47] Stewart DC , Maclure K , Bond CM , et al. Pharmacist prescribing in primary care: the views of patients across Great Britain who had experienced the service. Int J Pharm Pract. 2011;19:328‐332. 21899612 10.1111/j.2042-7174.2011.00130.x

[48] Taylor J , Krska J , Mackridge A . A community pharmacy‐based cardiovascular screening service: views of service users and the public. Int J Pharm Pract. 2012;20:277‐284. 22953766 10.1111/j.2042-7174.2012.00190.x

[49] Hill DR , Conroy S , Brown RC , Burt GA , Campbell D . Stakeholder views on pharmacist prescribing in addiction services in NHS Lanarkshire. J Subst Use. 2014;19:56‐67.

[50] MacLure K , George J , Diack L , Bond C , Cunningham S , Stewart D . Views of the Scottish general public on non‐medical prescribing. Int J Clin Pharm. 2013;35:704‐710. 23690252 10.1007/s11096-013-9792-x

[51] Anderson C , Thornley T . “It's easier in pharmacy”: why some patients prefer to pay for flu jabs rather than use the National Health Service. BMC Health Serv Res. 2014;14:35. 24456532 10.1186/1472-6963-14-35 PMC3902185

[52] Fakih S , Marriott JL , Boardman H , Anderson C , Hussainy SY . Comparing women pharmacy consumers’ experiences with weight loss treatment in Victoria and Nottingham: a cross‐sectional study. BMC Public Health. 2014;14:662. 24972611 10.1186/1471-2458-14-662 PMC4094681

[53] Heller R , Cameron ST . Evaluating the attractiveness of the availability of injectable progestogen contraceptives at the community pharmacy setting in the United Kingdom. Int J Pharm Pract. 2016;24:247‐252. 26875480 10.1111/ijpp.12249

[54] Fitzgerald N , Youngson E , Cunningham S , Watson M , Stewart D . Support for community pharmacy‐based alcohol interventions: a Scottish general public survey. Public Health. 2015;129:1431‐1438. 26296846 10.1016/j.puhe.2015.07.005

[55] Rodgers RM , Gammie SM , Loo RL , Corlett SA , Krska J . Comparison of pharmacist and public views and experiences of community pharmacy medicines‐related services in England. Patient Prefer Adherence. 2016;10:1749‐1758. 27672313 10.2147/PPA.S112931 PMC5026175

[56] Saramunee K , Dewsbury C , Cutler S , Mackridge AJ , Krska J . Public attitudes towards community pharmacy attributes and preferences for methods for promotion of public health services. Public Health. 2016;140:186‐195. 27481066 10.1016/j.puhe.2016.06.024

[57] Bissell P , Blenkinsopp A , Short D , Mason L . Patients’ experiences of a community pharmacy‐led medicines management service. Health Soc Care Community. 2008;16:363‐369. 18613912 10.1111/j.1365-2524.2007.00749.x

[58] Stewart DC , George J , Bond CM , Diack HL , McCaig DJ , Cunningham S . Views of pharmacist prescribers, doctors and patients on pharmacist prescribing implementation. Int J Pharm Pract. 2009;17:89‐94. 20214256

[59] Dhital R , Whittlesea CM , Norman IJ , Milligan P . Community pharmacy service users’ views and perceptions of alcohol screening and brief intervention. Drug Alcohol Rev. 2010;29:596‐602. 20973842 10.1111/j.1465-3362.2010.00234.x

[60] Hobson RJ , Scott J , Sutton J . Pharmacists and nurses as independent prescribers: exploring the patient's perspective. Fam Pract. 2010;27:110‐120. 19858124 10.1093/fampra/cmp070

[61] Lowrie R , Johansson L , Forsyth P , Bryce SL , McKellar S , Fitzgerald N . Experiences of a community pharmacy service to support adherence and self‐management in chronic heart failure. Int J Clin Pharm. 2014;36:154‐162. 24293306 10.1007/s11096-013-9889-2

[62] Tucker R , Stewart D . Why people seek advice from community pharmacies about skin problems. Int J Pharm Pract. 2015;23:150‐153. 24909457 10.1111/ijpp.12126

[63] Lindsey L , Husband A , Steed L , Walton R , Todd A . Helpful advice and hidden expertize: pharmacy users’ experiences of community pharmacy accessibility. J Public Health. 2017;39:609‐615. 10.1093/pubmed/fdw089 27591298

[64] Michie L , Cameron ST , Glasier A , Chen ZE , Milne D , Wilson S . Provision of contraception after emergency contraception from the pharmacy: evaluating the acceptability of pharmacy for providing sexual and reproductive health services. Public Health. 2016;135:97‐103. 26787315 10.1016/j.puhe.2015.11.017

[65] Mackridge AJ , Beynon CM , McVeigh J , Whitfield M , Chandler M . Meeting the health needs of problematic drug users through community pharmacy: a qualitative study. J Subst Use. 2010;15:367‐376.

[66] McCann LM , Haughey SL , Parsons C , et al. A patient perspective of pharmacist prescribing: ‘crossing the specialisms‐crossing the illnesses’. Health Expect. 2015;18:58‐68. 23067131 10.1111/hex.12008 PMC5060764

[67] Gidman W , Cowley J . A qualitative exploration of opinions on the community pharmacists’ role amongst the general public in Scotland. Int J Pharm Pract. 2013;21:288‐296. 23418884 10.1111/ijpp.12008

[68] Twigg MJ , Poland F , Bhattacharya D , Desborough JA , Wright DJ . The current and future roles of community pharmacists: views and experiences of patients with type 2 diabetes. Res Social Adm Pharm. 2013;9:777‐789. 23127392 10.1016/j.sapharm.2012.10.004

[69] Saramunee K , Krska J , Mackridge A , Richards J , Suttajit S , Phillips‐Howard P . How to enhance public health service utilization in community pharmacy?: general public and health providers’ perspectives. Res Social Adm Pharm. 2014;10:272‐284. 23089293 10.1016/j.sapharm.2012.05.006

[70] Wood K , Gibson F , Radley A , Williams B . Pharmaceutical care of older people: what do older people want from community pharmacy? Int J Pharm Pract. 2015;23:121‐130. 24905628 10.1111/ijpp.12127

[71] Baraitser P , Pearce V , Holmes J , Horne N , Boynton PM . Chlamydia testing in community pharmacies: evaluation of a feasibility pilot in south east London. Qual Saf Health Care. 2007;16:303‐307. 17693680 10.1136/qshc.2006.020883 PMC2464947

[72] Krska J , Mackridge AJ . Involving the public and other stakeholders in development and evaluation of a community pharmacy alcohol screening and brief advice service. Public Health. 2014;128:309‐316. 24713598 10.1016/j.puhe.2013.11.001

[73] Saramunee K , Krska J , Mackridge A , Richards J , Suttajit S , Phillips‐Howard P . General public's views on pharmacy public health services: current situation and opportunities in the future. Public Health. 2015;129:705‐715. 26008208 10.1016/j.puhe.2015.04.002

[74] Latif A , Boardman HF , Pollock K . Understanding the patient perspective of the English community pharmacy Medicines Use Review (MUR). Res Social Adm Pharm. 2013;9:949‐957. 23506650 10.1016/j.sapharm.2013.01.005

[75] Tinelli M , Ryan M , Bond C . Patients’ preferences for an increased pharmacist role in the management of drug therapy. Int J Pharm Pract. 2009;17:275‐282. 20214269

[76] Porteous T , Ryan M , Bond C , Watson M , Watson V . Managing minor ailments; The public's preferences for attributes of community pharmacies. A discrete choice experiment. PLoS ONE. 2016;11:e0152257. 27031588 10.1371/journal.pone.0152257 PMC4816534

[77] Mansell K , Perepelkin J . Patient awareness of specialized diabetes services provided in community pharmacies. Res Social Adm Pharm. 2011;7:396‐405. 21272537 10.1016/j.sapharm.2010.10.004

[78] Smith M , Cannon‐Breland ML , Spiggle S . Consumer, physician, and payer perspectives on primary care medication management services with a shared resource pharmacists network. Res Social Adm Pharm. 2014;10:539‐553. 24055137 10.1016/j.sapharm.2013.08.003

[79] McMillan SS , Sav A , Kelly F , King MA , Whitty JA , Wheeler AJ . How to attract them and keep them: the pharmacy attributes that matter to Australian residents with chronic conditions. Int J Pharm Pract. 2014;22:238‐245. 24134587 10.1111/ijpp.12075

[80] Um IS , Armour C , Krass I , Gill T , Chaar BB . Consumer perspectives about weight management services in a community pharmacy setting in NSW, Australia. Health Expect. 2014;17:579‐592. 22646843 10.1111/j.1369-7625.2012.00788.x PMC5060745

[81] Jin X , Azhar S , Murtaza G , et al. Quantitative study evaluating perception of general public towards role of pharmacist in health care system of Pakistan. Acta Pol Pharm. 2014;71:869‐875. 25362816

[82] El Hajj MS , Salem S , Mansoor H . Public's attitudes towards community pharmacy in Qatar: a pilot study. Patient Prefer Adherence. 2011;5:405‐422. 21949604 10.2147/PPA.S22117 PMC3176180

[83] Mathialagan A , Nagalinggam P , Mathialagan S , Kirby BP . Relationship between performance barriers and pharmacist competency towards the implementation of an expanded public health pharmacy role. Int J Pharm Pract. 2015;23:320‐326. 25582973 10.1111/ijpp.12170

[84] Carter SR , Moles R , White L , Chen TF . Patients’ willingness to use a pharmacist‐provided medication management service: the influence of outcome expectancies and communication efficacy. Res Social Adm Pharm. 2012;8:487‐498. 22381916 10.1016/j.sapharm.2012.01.002

[85] Knox K , Kelly F , Mey A , Hattingh L , Fowler JL , Wheeler AJ . Australian mental health consumers’ and carers’ experiences of community pharmacy service. Health Expect. 2015;18:2107‐2120. 24612376 10.1111/hex.12179 PMC5810739

[86] Tan CL , Hassali MA , Saleem F , Shafie AA , Aljadhay H , Gan VB . Building intentions with the theory of planned behaviour: a qualitative assessment of salient beliefs about pharmacy value added services in Malaysia. Health Expect. 2016;19:1215‐1225. 26426210 10.1111/hex.12416 PMC5139050

[87] Palombi L , Kading M , Hayes C . The public health pharmacist and the role of the pharmacy curriculum: a call to action. Curr Pharm Teach Learn. 2013;5:477‐482.

[88] Jesson J , Bissell P . Public health and pharmacy: a critical review. Crit Public Health. 2006;16:159‐169.

[89] Ogunbayo OJ , Schafheutle EI , Cutts C , Noyce PR . A qualitative study exploring community pharmacists’ awareness of, and contribution to, self‐care support in the management of long‐term conditions in the United Kingdom. Res Social Adm Pharm. 2015;11:859‐879. 25677228 10.1016/j.sapharm.2014.12.010

[90] Latif A , Waring J , Watmough D , et al. Examination of England's New Medicine Service (NMS) of complex health care interventions in community pharmacy. Res Social Adm Pharm. 2016;12:966‐989. 26806858 10.1016/j.sapharm.2015.12.007

[91] Vella M , Grima M , Wirth F , et al. Consumer perception of community pharmacist extended professional services. J Pharm Health Serv Res. 2015;6:91‐96.

[92] Carter BL , Bergus GR , Dawson JD , et al. A cluster randomized trial to evaluate physician/pharmacist collaboration to improve blood pressure control. J Clin Hypertens (Greenwich). 2008;10:260‐271. 18401223 10.1111/j.1751-7176.2008.07434.x PMC2453045

[93] Kiel PJ , McCord AD . Pharmacist impact on clinical outcomes in a diabetes disease management program via collaborative practice. Ann Pharmacother. 2005;39:1828‐1832. 16219894 10.1345/aph.1G356

[94] Hunt JS , Siemienczuk J , Pape G , et al. A randomized controlled trial of team‐based care: impact of physician‐pharmacist collaboration on uncontrolled hypertension. J Gen Intern Med. 2008;23:1966‐1972. 18815843 10.1007/s11606-008-0791-x PMC2596500

[95] Carter BL , Ardery G , Dawson JD , et al. Physician and pharmacist collaboration to improve blood pressure control. Arch Intern Med. 2009;169:1996‐2002. 19933962 10.1001/archinternmed.2009.358 PMC2882170

[96] Hatah E , Braund R , Duffull S , Tordoff J . General practitioners’ perceptions of pharmacists’ new services in New Zealand. Int J Clin Pharm. 2012;34:364‐373. 22359183 10.1007/s11096-012-9617-3

[97] Dhillon AK , Hattingh HL , Stafford A , Hoti K . General practitioners’ perceptions on home medicines reviews: a qualitative analysis. BMC Fam Pract. 2015;16:16. 25881287 10.1186/s12875-015-0227-8 PMC4332443

[98] McGrath SH , Snyder ME , Duenas GG , Pringle JL , Smith RB , McGivney MS . Physician perceptions of pharmacist‐provided medication therapy management: qualitative analysis. J Am Pharm Assoc (2003). 2010;50:67‐71. 20097641 10.1331/JAPhA.2010.08186

[99] Hughes CM , McCann S . Perceived interprofessional barriers between community pharmacists and general practitioners: a qualitative assessment. Br J Gen Pract. 2003;53:600‐606. 14601335 PMC1314673

[100] Torjesen I . More than 400 pharmacists will be recruited to GP surgeries by next year. BMJ. 2015;351:h6167. 26572158 10.1136/bmj.h6167

[101] England NHS . General Practice Forward View (GPFV)‐Clinical Pharmacists in General Practice Phase 2 Guidance for Applicants. London: NHS England; 2016:1‐19.

[102] England NHS . General Practice Forward View. London: NHS England; 2016.

[103] Bishop AC , Boyle TA , Morrison B , et al. Public perceptions of pharmacist expanded scope of practice services in Nova Scotia. Can Pharm J (Ott). 2015;148:274‐283. 26445585 10.1177/1715163515596757 PMC4561456

[104] Garcia GM , Snyder ME , McGrath SH , Smith RB , McGivney MS . Generating demand for pharmacist‐provided medication therapy management: identifying patient‐preferred marketing strategies. J Am Pharm Assoc (2003). 2009;49:611‐616. 19748867 10.1331/JAPhA.2009.08089

[105] Department of Health . Improving Patients’ Access to Medicines: A Guide to Implementing Nurse and Pharmacist Independent Prescribing Within the NHS in England. London: Department of Health; 2006.

[106] PSNC . Summary Care Record access to be rolled out to community pharmacies. http://psnc.org.uk/our-news/summary-care-record-access-to-be-rolled-out-to-community-pharmacies/. Accessed January 15, 2017.

