# Supplementary material for: Patient and public perspectives of community pharmacies in the United Kingdom: A systematic review
Source: Health Expect. 2017 Nov 8;21(2):409–28. doi: 10.1111/hex.12639 (PMC5867331; doi:10.1111/hex.12639)
Supplement: Supplementary file 1 [file HEX-21-409-s001.docx]

# Appendix S1: Search strategy for each database

| **Embase** | | |
| --- | --- | --- |
| Search | Query | Items found |
| #1 | Awareness (mapped to awareness) OR “patient preference?’’ (mapped to patient preference OR patient attitude) OR view? OR perspective? OR access (access to information OR healthcare access) OR opinion? OR perception? | 1524669 |
| #2 | Patient? (mapped to patient) OR “service user?’’ OR consumer? (mapped to consumer) OR public? (mapped to public opinion) OR customer? OR client? | 8645684 |
| #3 | “Community pharmac*” (mapped to community pharmacy) | 19398 |
| #4 | Service? ( mapped to National Health service OR mental health service OR Health service OR preventive health service OR public health service) OR “pharmaceutical care’’ (mapped to pharmaceutical care OR Patient care) OR “public health’’ (mapped to public health) OR “medication therapy management’’ (mapped to medication therapy management) OR “long term care’’ (mapped to long term care) | 1452809 |
| #5 | #1 AND #2 AND #3 AND #4 | 2313 |

- *2005-Present filter activated for all searches*
- *Mapping= matches terms with the controlled vocabulary of the database*

| **PubMed** | | |
| --- | --- | --- |
| Search | Query | Items found |
| #1 | awareness[MeSH Terms] OR awareness OR patient preference[MeSH Terms] OR “patient preference” OR “patient preferences” OR view* OR perspective* OR access OR public opinion[MeSH Terms] OR opinion* OR perceptions[MeSH Terms] OR perception* | 772285 |
| #2 | patients[MeSH Terms] OR patient OR patients OR “service user” OR “service users” OR consumer* OR public* OR customer* OR client[MeSH Terms]) OR client* | 3391550 |
| #3 | community pharmacy[MeSH Terms]) OR “community pharmacy” OR community pharmacies [MeSH Terms]) OR “community pharmacies” OR “community pharmacist” OR “community pharmacists” | 5467 |
| #4 | Service* OR pharmaceutical care[MeSH Terms] OR “pharmaceutical care” OR public health[MeSH Terms] OR “public health” OR medication therapy management[MeSH Terms] OR “medication therapy management” OR long term care[MeSH Terms] OR “long term care” | 3547068 |
| #5 | #1 AND #2 AND #3 AND #4 | 1134 |

- *Filters for all searches: Publication date from 2005/01/01 to 2016/12/31*
- *MeSH: Medical subject heading; a comprehensive controlled vocabulary*

| **Scopus** | | |
| --- | --- | --- |
| Search | Query | Items found |
| *#1* | TITLE-ABS-KEY (awareness ) OR ( “patient preference”) OR (view) OR (perspective) OR (access) OR (opinion) OR (perception) AND DOCTYPE ( ar OR re ) AND PUBYEAR > 2004 | *1,531,743* |
| *#2* | TITLE-ABS-KEY (patient) OR ( “service user”) OR (consumer) OR (public) OR (customer) OR (client) AND DOCTYPE ( ar OR re ) AND PUBYEAR > 2004 | *3,820,453* |
| *#3* | TITLE-ABS-KEY (“community pharmac*”) AND DOCTYPE ( ar OR re ) AND PUBYEAR > 2004 | *4,926* |
| *#4* | TITLE-ABS-KEY (Service) OR (“pharmaceutical care”) OR (“public health”) OR (“medication therapy management”) OR( “long term care”) AND DOCTYPE ( ar OR re ) AND PUBYEAR > 2004 | *887,872* |
| *#5* | #1 AND #2 AND #3 AND #4 | *1,069* |

- *DOCTYPE (ar or re) = document type either Article or Review*
- *Timeline: 2005-present activated for all searches*
- *Scopus automatically searches for plurals*

| **Web of Science** | | |
| --- | --- | --- |
| Search | Query | Items found |
| *#1* | Awareness OR “patient preferences” OR “patient preference” OR view? OR perspective? OR access OR opinion? OR perception? | 841,902 |
| *#2* | Patient? OR “service user” OR “service user” OR consumer? OR public? OR customer? OR client? | 2,705,191 |
| *#3* | “community pharmac*” | 3,791 |
| *#4* | Service? OR “pharmaceutical care” OR “public health” OR “medication therapy management” OR “long term care” | 440,977 |
| *#5* | #1 AND #2 AND #3 AND #4 | 413 |

- *Timespan 2005-2016 activated for all searches*

| **International Pharmaceutical Abstracts** | | |
| --- | --- | --- |
| Search | Query | Items found |
| #1 | Awareness (mapped to awareness) OR “patient preference?’’ (mapped to patient preference OR patient attitude) OR view? OR perspective? OR access (access to information OR healthcare access) OR opinion? OR perception? | 23,587 |
| #2 | Patient? (mapped to patient) OR “service user?’’ OR consumer? (mapped to consumer) OR public? (mapped to public opinion) OR customer? OR client? | 213,853 |
| #3 | “Community pharmac*” (mapped to community pharmacy) | 2003 |
| #4 | Service? ( mapped to National Health service OR mental health service OR Health service OR preventive health service OR public health service) OR “pharmaceutical care’’ (mapped to pharmaceutical care OR Patient care) OR “public health’’ (mapped to public health) OR “medication therapy management’’ (mapped to medication therapy management) OR “long term care’’ (mapped to long term care) | 40,170 |
| #5 | #1 AND #2 AND #3 AND #4 | 333 |

- *2005-present filter activated for all searches*
- *Mapping= Matches terms with the controlled vocabulary of a database*

| **Science Direct** | | |
| --- | --- | --- |
| Search | Query | Items found |
| *#1* | *TITLE-ABSTR-KEY(Awareness) OR (“patient preference”) OR (view) OR (perspective) OR*  *(access)OR (opinion) OR (perception)* | *315,735* |
| *#2* | TITLE-ABSTR-KEY (Patient) OR (“service user”) OR (consumer) OR (public) OR (customer) OR (client) | *990,633* |
| *#3* | TITLE-ABSTR-KEY (“community pharmac*”) | *1,237* |
| *#4* | (Service) OR (“pharmaceutical care”) OR (“public health”) OR (“medication therapy management”) OR (“long term care”) | *135,568* |
| *#5* | #1 AND #2 AND #3 AND #4 | **179** |

- *Journal tab was applied for all searches*
- *2005-present filter activated for all searches*
- *Using the singular form of a word finds the singular, plural, and possessive forms of most words.*

| **CINAHL Plus** | | |
| --- | --- | --- |
| Search | Query | Items found |
| *#1* | Awareness OR “patient preference” OR “patient preferences” OR view OR perspective OR access OR opinion OR perception | 245,804 |
| *#2* | Patient OR “service user” OR “service users” OR consumer OR public or customer OR client | 1,053,015 |
| *#3* | “Community pharmac*” | 1,614 |
| *#4* | Service OR “pharmaceutical care” OR “public health” OR “medication therapy management” OR “long term care” | 339,824 |
| *#5* | #1 AND #2 AND #3 AND #4 | 154 |

- *Timeline 2005-2016 applied for all searches*
- *When a singular word is searched, the plural and possessive forms of that word will also be searched*

| **PsycINFO** | | |
| --- | --- | --- |
| Search | Query | Items found |
| #1 | Awareness (mapped to awareness) OR “patient preference?’’ (mapped to client attitudes) OR view? OR perspective? OR access OR opinion? (mapped to attitude) OR perception? | 993149 |
| #2 | Patient? (mapped to patients) OR “service user?’’ OR consumer? OR public? OR customer? OR client? (mapped to clients) | 924353 |
| #3 | “Community pharmac*” | 551 |
| #4 | Service? OR “pharmaceutical care’’ (mapped to healthcare services) OR “public health’’ (mapped to public health) OR “medication therapy management’’ OR “long term care’’ (mapped to long term care) | 320307 |
| #5 | #1 AND #2 AND #3 AND #4 | 104 |

- *2005-present filter activated for all searches*
