# Supplementary material for: Patient and public perspectives of community pharmacies in the United Kingdom: A systematic review
Source: Health Expect. 2017 Nov 8;21(2):409–28. doi: 10.1111/hex.12639 (PMC5867331; doi:10.1111/hex.12639)
Supplement: Supplementary file 2 [file HEX-21-409-s002.docx]

Appendix S2: Critical appraisal using the nine item checklist by Hawker et al.

**Good= 4 points, Fair= 3 points, Poor= 2 points, Very poor= 1 point*

| Study | 1. Title and abstract | 2. Introduction and aims | 3. Method and data | 4. Sampling | 5. Data analysis | 6. Ethics and bias | 7. Results | 8. Transferability and generalizability | 9. Implications and usefulness | Total score  (max: 36) |
| --- | --- | --- | --- | --- | --- | --- | --- | --- | --- | --- |
| Baraitser et al. 2007 ^71^ | 3 | 2 | 3 | 3 | 3 | 1 | 3 | 2 | 4 | 24 |
| Tinelli et al. 2007^43^ | 4 | 2 | 4 | 3 | 4 | 2 | 4 | 3 | 3 | 29 |
| Bissell et al. 2008^57^ | 3 | 4 | 4 | 3 | 4 | 2 | 4 | 3 | 3 | 30 |
| Stewart et al. 2008^44^ | 4 | 4 | 2 | 3 | 2 | 3 | 3 | 2 | 3 | 26 |
| Stewart et al. 2009^58^ | 3 | 4 | 3 | 3 | 2 | 2 | 4 | 3 | 4 | 28 |
| Tinelli et al. 2009^75^ | 3 | 3 | 4 | 4 | 4 | 2 | 4 | 3 | 3 | 30 |
| Dhital et al. 2010^59^ | 3 | 4 | 3 | 4 | 3 | 2 | 3 | 3 | 4 | 29 |
| Hobson et al. 2010^60^ | 4 | 3 | 3 | 3 | 4 | 2 | 4 | 3 | 4 | 30 |
| Krska et al. 2010^45^ | 3 | 3 | 3 | 3 | 3 | 2 | 3 | 2 | 3 | 25 |
| Krska & Morecroft, 2010^46^ | 3 | 4 | 4 | 2 | 2 | 2 | 3 | 2 | 3 | 25 |
| Mackridge et al. 2010^65^ | 3 | 3 | 3 | 2 | 2 | 2 | 2 | 3 | 2 | 22 |
| Stewart et al. 2011^47^ | 4 | 4 | 2 | 3 | 2 | 3 | 3 | 2 | 3 | 26 |
| Taylor et al. 2012^48^ | 3 | 4 | 3 | 3 | 3 | 2 | 4 | 2 | 4 | 28 |
| Gidman and Cowley, 2013^67^ | 4 | 3 | 4 | 4 | 4 | 2 | 4 | 4 | 4 | 33 |
| Latif et al. 2013^74^ | 4 | 4 | 4 | 3 | 4 | 2 | 4 | 3 | 4 | 32 |
| Maclure et al. 2013^50^ | 3 | 3 | 3 | 2 | 3 | 3 | 3 | 3 | 3 | 26 |
| Twigg et al. 2013^68^ | 4 | 4 | 4 | 4 | 4 | 2 | 4 | 4 | 4 | 34 |
| Anderson & Thornley, 2014^51^ | 3 | 3 | 2 | 3 | 1 | 3 | 3 | 2 | 3 | 23 |
| Fakih et al. 2014^52^ | 4 | 4 | 3 | 3 | 4 | 3 | 4 | 3 | 3 | 31 |
| Hill et al. 2014^49^ | 3 | 3 | 3 | 2 | 1 | 2 | 3 | 2 | 3 | 22 |
| Krska and Mackridge, 2014^72^ | 4 | 4 | 3 | 2 | 2 | 2 | 4 | 2 | 3 | 26 |
| Lowrie et al. 2014^61^ | 4 | 2 | 3 | 3 | 4 | 2 | 4 | 2 | 3 | 27 |
| Saramunee et al. 2014 ^69^ | 4 | 3 | 4 | 3 | 4 | 2 | 4 | 4 | 4 | 32 |
| Tucker & Stewart, 2014^62^ | 3 | 2 | 3 | 3 | 2 | 2 | 2 | 3 | 2 | 22 |
| Fitzgerald et al. 2015^54^ | 4 | 3 | 3 | 4 | 4 | 2 | 4 | 3 | 4 | 31 |
| McCann et al. 2015^66^ | 4 | 4 | 4 | 3 | 3 | 2 | 4 | 3 | 3 | 30 |
| Saramunee et al. 2015 ^73^ | 3 | 4 | 4 | 3 | 3 | 2 | 4 | 2 | 3 | 28 |
| Wood et al. 2015^70^ | 4 | 2 | 4 | 4 | 4 | 2 | 4 | 4 | 3 | 31 |
| Heller and Cameron, 2016^53^ | 4 | 3 | 3 | 3 | 3 | 2 | 4 | 2 | 2 | 26 |
| Lindsey et al. 2016^63^ | 3 | 3 | 4 | 4 | 4 | 3 | 4 | 3 | 3 | 31 |
| Michie et al. 2016^64^ | 4 | 3 | 3 | 3 | 2 | 2 | 3 | 2 | 3 | 25 |
| Porteous et al. 2016 ^76^ | 4 | 4 | 3 | 4 | 4 | 4 | 3 | 3 | 3 | 32 |
| Rodgers et al. 2016^55^ | 4 | 4 | 3 | 3 | 4 | 3 | 4 | 3 | 3 | 31 |
| Saramunee et al. 2016^56^ | 4 | 3 | 4 | 4 | 4 | 2 | 4 | 4 | 3 | 32 |
| Mean±SD | 3.6 ± 0.5 | 3.3 ± 0.7 | 3.3 ± 0.6 | 3.1 ± 0.6 | 3.3 ± 1.0 | 2.2 ± 0.6 | 3.6 ± 0.6 | 2.8 ± 0.7 | 3.2 ± 0.6 | 28.1 ± 3.4 |

**1. Abstract and title: Did they provide a clear description of the study? 2. Introduction and aims: Was there a good background and clear statement of the aims of the research? 3. Method and data: Is the method appropriate and clearly explained?*

*4. Sampling: Was the sampling strategy appropriate to address the aims? 5. Data analysis: Was the description of the data analysis sufficiently rigorous? 6. Ethics and bias: Have ethical issues been addressed, and what has necessary ethical approval gained? Has the relationship between researchers and participants been adequately considered? 7. Results: Is there a clear statement of the findings? 8. Transferability or generalizability: Are the findings of this study transferable (generalizable) to a wider population? 9. Implications and usefulness: How important are these findings to policy and practice?*
